# Using a stochastic continuous-time Markov chain model to examine alternative timing and duration of the COVID-19 lockdown in Kuwait: what can be done now?

**DOI:** 10.1186/s13690-021-00778-y

**Published:** 2022-01-08

**Authors:** Mustafa Al-Zoughool, Tamer Oraby, Harri Vainio, Janvier Gasana, Joseph Longenecker, Walid Al Ali, Mohammad AlSeaidan, Susie Elsaadany, Michael G. Tyshenko

**Affiliations:** 1 Department of Environmental and Occupational Health Faculty of Public Health, University of Kuwait, Kuwait City, Kuwait; 2 School of Mathematical and Statistical Sciences, University of Texas Rio Grande Valley, Edinburg, TX 78539 USA; 3 Department of Epidemiology and Biostatistics, Faculty of Public Health, University of Kuwait, 13110 Safat, Kuwait; 4 Department of Occupational Health, Ministry of Health, Kuwait City, Kuwait; 5 Department of Pathology and Laboratory Medicine, Faculty of Medicine, University of Ottawa, Ottawa, ON K1H 8M5 Canada; 6 McLaughlin Centre for Population Health Risk Assessment, Faculty of Medicine, University of Ottawa, Ottawa, ON K1N 6N5 Canada

**Keywords:** Kuwait, COVID-19, Stochastic model, Lockdown timing, Lockdown duration, Actual incidence, Hospitalization

## Abstract

**Background:**

Kuwait had its first COVID-19 in late February, and until October 6, 2020 it recorded 108,268 cases and 632 deaths. Despite implementing one of the strictest control measures-including a three-week complete lockdown, there was no sign of a declining epidemic curve. The objective of the current analyses is to determine, hypothetically, the optimal timing and duration of a full lockdown in Kuwait that would result in controlling new infections and lead to a substantial reduction in case hospitalizations.

**Methods:**

The analysis was conducted using a stochastic Continuous-Time Markov Chain (CTMC), eight state model that depicts the disease transmission and spread of SARS-CoV 2. Transmission of infection occurs between individuals through social contacts at home, in schools, at work, and during other communal activities.

**Results:**

The model shows that a lockdown 10 days before the epidemic peak for 90 days is optimal but a more realistic duration of 45 days can achieve about a 45% reduction in both new infections and case hospitalizations.

**Conclusions:**

In the view of the forthcoming waves of the COVID19 pandemic anticipated in Kuwait using a correctly-timed and sufficiently long lockdown represents a workable management strategy that encompasses the most stringent form of social distancing with the ability to significantly reduce transmissions and hospitalizations.

**Supplementary Information:**

The online version contains supplementary material available at 10.1186/s13690-021-00778-y.

## Background

On December 312,019, A cluster of viral-related pneumonia cases was identified in Wuhan, China. These cases were found to be caused by a new respiratory betacoronavirus [1, 2] later renamed to Severe Acute Respiratory Syndrome Coronavirus 2 (SARS-CoV-2) [3]. The resulting constellation of respiratory symptoms caused by SARS-CoV-2 was given the designation of COVID-19 (coronavirus disease 2019). Travel related cases from China seeded outbreaks in several other countries. The World Health Organization (WHO) declared a Public Health Emergency of International Concern on January 30, 2020 [4]. Shortly afterwards, the number of diagnosed cases increased several-fold and the number of countries which reported cases increased, the WHO declared the outbreak as global pandemic on March 11, 2020 [5].

During the early phases of a pandemic Public Health officials must make decision given a high degree of uncertainty. The degree, type and timing of interventions to reduce the health burden of a pandemic must be applied using a precautionary approach with little epidemiological evidence. Many countries affected by growing numbers of COVID-19 cases shifted management of the pandemic from contact tracing, travel restrictions and quarantine of case contacts [2] to include broader population interventions such as public health communications, social distancing (school closures, work from home), voluntary quarantines (self-isolation), curfews, limited cross-border or regional travel, and lockdowns to reduce person-to-person transmission [6].

Social distancing reduces transmission between people where individuals may be asymptomatic but infectious. Evidence suggests that social distancing as a strategy can be effective to attenuate the magnitude of a pandemic [7]. Social distancing spans a continuum from individuals who voluntarily social distance while interacting in the community, self-isolating at home, entering hospital isolation or quarantine, to more complex community-wide quarantines and involuntary lockdowns that enforce major movement restrictions at a population level [7]. The most stringent form of social distancing is the use of a community-wide quarantine or “lockdown” that can be applied to a city, region or entire country as a way to drastically stop the movement of people.

Mathematical modelling can help inform decision-making of lockdowns and provide an evidence base for rational pandemic management. Herein, we use Kuwait as a case study to show the effects of different timings and lengths of hypothetical lockdown scenarios. Kuwait has a relatively small area and population with unique demographic characteristics that make it of interest to model the effects of potential interventions. Using a stochastic Continuous-Time Markov Chain (CTMC) model we analyzed the effects of lockdown timing in Kuwait due to COVID-19 with emphasis on the attack rates, and peak hospitalizations.

In addition, the model investigates the effect of various hypothetical timings (days before the epidemic peak) and durations (length in days) of the full lockdown. The model includes country-specific social contact matrices [8] and considers environmental transmission parameters. Environmental transmission occurs through coming in contact with surfaces or fomites that have been contaminated with the virus. This route of exposure has been reported with SARS and MERS-CoV-1 viruses previously [9]. A recent report showed that after application, the SARS-CoV-2 virus remained viable experimentally in aerosols for 3 h, on copper surfaces up to 4 h, on cardboard up to 24 h, and on plastic and stainless steel up to 72 h (depending on the amount of inoculum shed) [10].

The main purpose of the current analysis is to investigate hypothetical lockdown scenarios and help to inform Kuwait’s pandemic management strategy for future COVID-19 waves.

## Methods

### Model description

**Fig. 1:**
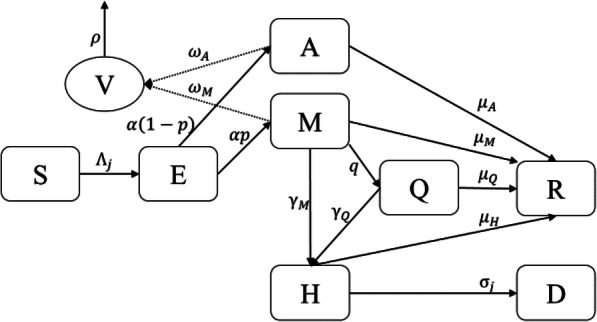
Schematic digraph of transitions of individuals between compartments in which transmission and transition rates are indicated over the arrows. See Table S3 for definition of model’s parameters. The force of infection *Λ*_*j*_ is given in eq. (1), which depend on the environmental contact matrix *(C*^*V*^*)* and social contact matrices *(C)* for school, work, household, and others

Previously we developed a CTMC model with eight states to depict the disease transmission and spread of SARS-CoV 2: susceptible (S), exposed (E), infected but asymptomatic (A), mildly infected and symptomatic (M), severely infected, symptomatic and hospitalized (H), detected and quarantined (Q), recovered (R), and dead (D) (SEAMHQRD-V) [11]. A CTMC can capture the initial disease dynamics and accommodate the uncertainties involved in the disease transmission process. It is also recommended given the small number of cases usually encountered during the beginning of an epidemic. The model also considers the environmental transmission denoted by V (see Fig. 1).

We split the population age groups in to three compartments: children (0–18 years), adults (19–64 year) and seniors (65 years or older), designated as *N*_*c*_, *N*_*a*_, *and N*_*s*_.

We assume an initial random number of individuals in the E, A, and I compartment given by e_0, a_0, and i_0, respectively. The probability of transitions of the CTMC, X(t), from state x to state y (denoted by x → y) in the interval (t,t + dt) for very small dt is given by

$$ P\left(\boldsymbol{X}\left(t+ dt\right)=y|\boldsymbol{X}(t)=x\right)={r}_{xy}\  dt+o(dt) $$

The disease transitions in the underlying CTMC model are occurring according to the descriptions and rates given in the Appendix-Table 1.

Transmission of infection between individuals is assumed to occur through social contacts that occur at home, in school, at work, and during other activities (e.g. shopping). The social contact matrices used in the CTMC model for Kuwait were adapted from the study by [8] which estimated age- and location-specific contact patterns for 144 countries. Perm et al. (2017) extrapolated results from an earlier population-based contact diaries in eight European countries in the POLYMOD study using a Bayesian hierarchical model [12]. Here, we use the resulting five social-contact matrices specific to Kuwait ***C***^*k*^ for *k* = *sc* (school), *h* (household), *w* (work), *o* (other), and *v* (environment). We define the contact rates between the three age groups:

1 $$ {\boldsymbol{C}}^k=\left(\begin{array}{ccc}{C}_{cc}^k& {\mathrm{C}}_{ca}^k& {C}_{cs}^k\\ {}{C}_{ac}^k& {C}_{aa}^k& {C}_{as}^k\\ {}{C}_{sc}^k& {C}_{sa}^k& {C}_{ss}^k\end{array}\right) $$

The home contact matrix was normalized by the number of household members in each age group [13].

A socially altered contact matrix $\overset{\sim }{\boldsymbol{C}}$ defines the contact rates between the three age groups with social distances and lockdown, where the column vector, the entries of $\overset{\sim }{\boldsymbol{C}}$ are defined by

$$ {\overset{\sim }{C}}_{ij}={C}_{ij}^h+\sum \limits_{k= sc,w,o}{C}_{ij}^k\left(1-\tilde{p}_{j}^k(t)\right)\left(1-\tilde{p}_{i}^k(t)\right) $$

And $$ {\overset{\sim }{C}}_{ij}^v={C}_{ij}^v\left(1-\tilde{p}_{i}^v(t)\right) $$

for *i* and *j* = *c*, *a*, *s*. The vector $\left({p}_c^k,{p}_a^k,{p}_s^k\right)$ defines the proportion of those practicing social distances in the three age groups at the different location types *k* = *sc*, *w*, *o*, *v*, and so degree of adherence to social distancing. The parameter $\tilde{p}_{i}^k$ is the government imposed closures and enforced stay-home which only takes values $\tilde{p}_{i}$ based on a policy turning points *t*_*l*, *i*_ in a way that $\tilde{p}_{i}^k(t)={p}_i^k\ I\left({t}_{1,i}\ge t\ge {t}_{0,i}\right)$. During the full lockdown, social contact matrix outside households is reduced to zero which in turn leads to increase in household contacts.

*The environmental transmission has the component at time* t *is* V_j_ for *j* = *c*, *a*, and *s*, where

$$ {\overset{\sim }{\upomega}}_{\mathrm{A},\mathrm{i}}(t)={\upomega}_{\mathrm{A}}\left(1-\tilde{p}_{i}^v(t)\right) $$

$$ {\overset{\sim }{\upomega}}_{\mathrm{M},\mathrm{i}}(t)={\upomega}_{\mathrm{M}}\left(1-\tilde{p}_{i}^v(t)\right) $$

and where ω_A_ *(*ω_M_) *is the number of individuals equivalent to environmental contamination/deposit made by asymptomatic (mildly infected) individual per place.*

Different epidemiological measures and their statistics are simulated from the CTMC. The first measure of the actual incidence is defined as the proportion of the newly infected individuals to the population every day over the course of the epidemic. The second measure is the total attack rate which is the fraction of people that contract the disease in an at-risk population over the epidemic period. The third measure is hospital case load, defined as the fraction of the total population size that is hospitalized for COVID-19 treatment at any given time.

### Extension of the model and model parameterization

Model assumptions are listed in Table 2 of the Appendix. Also, the list of parameters used in the model, their description and values is shown in the Appendix-Table 3. We further use parameter functional of the parameters to reflect the different disease control and mitigation measures.

### Model calibration

A *R*_0_ formula of the CTMC was determined in Oraby et al. (2020) [11] using an approximation of the CTMC by a multi-type branching process to be proportional to the spectral radius (***ρ***) of a simple transformation of the contact matrix $\overset{\sim }{\boldsymbol{C}}$. That is,

2 $$ {\mathrm{R}}_0=\boldsymbol{\rho} \left(\mathbf{B}\right)\left[\left(1-p\right)\frac{1+r\ {\overset{\sim }{\upomega}}_{\mathrm{A}}/\rho }{\upmu_{\mathrm{A}}}+p\ \frac{1+r\ {\overset{\sim }{\upomega}}_{\mathrm{M}}/\rho }{q+{\upmu}_{\mathrm{M}}+{\upgamma}_M}\right] $$

where

3 $$ \mathbf{B}=\left(\begin{array}{ccc}{\beta}_c{N}_c& 0& 0\\ {}0& {\beta}_a{N}_a& 0\\ {}0& 0& {\beta}_s{N}_s\end{array}\right)\overset{\sim }{\boldsymbol{C}}\left(\begin{array}{ccc}\frac{1}{N_c}& 0& 0\\ {}0& \frac{1}{N_a}& 0\\ {}0& 0& \frac{1}{N_s}\end{array}\right) $$

We use that formula of *R*_0_ to calibrate the probability of transmission to different age-groups. We use a value of *R*_0_ = 6.47 as estimated in Tang et al. (2020) [14] by a very close model for data from Wuhan city.

We used the tau-leap method [15] to simulate the stochastic CTMC model for 1000 iterations. It is known that the size of the epidemic has a chance to be zero in CTMC models [16], which we exclude given that attack rates cannot, epidemiologically, have a value of zero and the COVID-19 virus has already demonstrated a significant potential to spread between individuals. Results of these 1000 simulations are presented in Figs. 3 and 4 as gray shaded band with the solid line representing the average of these simulations.

## Results

**Fig. 2:**
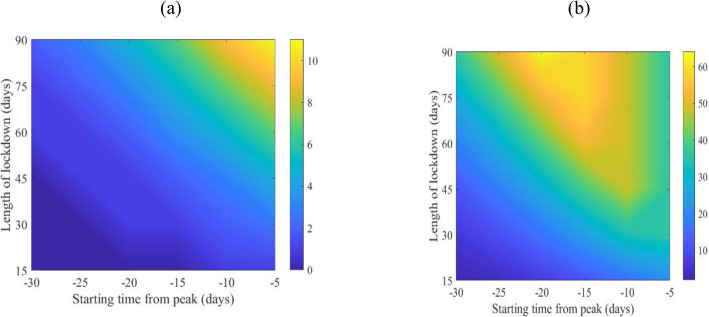
Mean of percentage reduction in Kuwait of the attack rates and peak of hospitalization for the COVID-19. The bar to the right of the figures represents percent reduction

Figure 2 shows the effect of the timing (days before the epidemic peak) and duration (days of implementation) of the lockdown on attack rates, and hospitalization. Figure 2a shows that implementation of the lockdown 5–10 days before the epidemic peak for 90 days duration was associated with the maximum reduction in attack rates of about 10%. Figure 2b shows that peak hospitalizations were reduced by nearly 60% when the lockdown was implemented around 15 days before the peak of the epidemic, and this lockdown would last for 90 days.

The same figure also shows that implementing the lockdown that is half as long (45 days) can still achieve about a 50% reduction in hospitalizations. However, the timing “window” to start the lockdown to achieve the reduction under the 45-day lockdown is shorter in duration (3–4 days) and would require more precision to time the start of the lockdown event compared to longer lockdown durations.

Thus, hypothetically, a lockdown started 15 days before the epidemic peak for a duration of 90 days would likely achieve the maximum benefit in terms of hospitalization case load reduction which is an indicator of the spread of the virus among vulnerable groups.

**Fig. 3:**
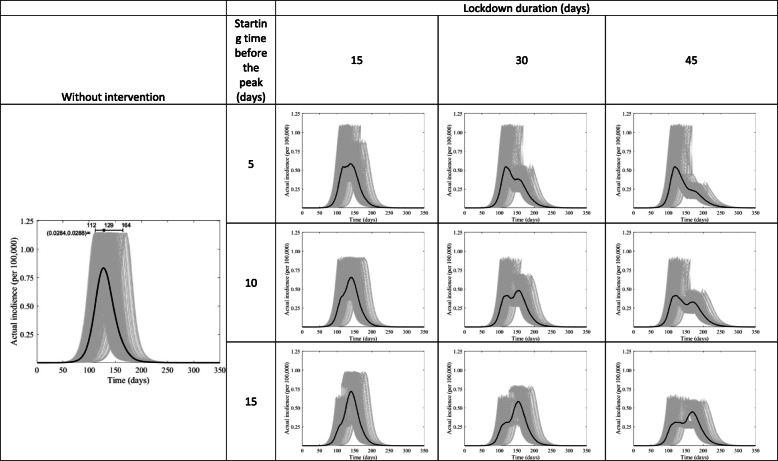
The course of the actual COVID-19 incidence in Kuwait if the lockdown starts 5, 10, or 15 days before the peak and lasts for 15, 30, or 45 days. The uncertainty is shown in the gray shaded areas, while the solid black curve shows the mean of the simulation results

**Fig. 4:**
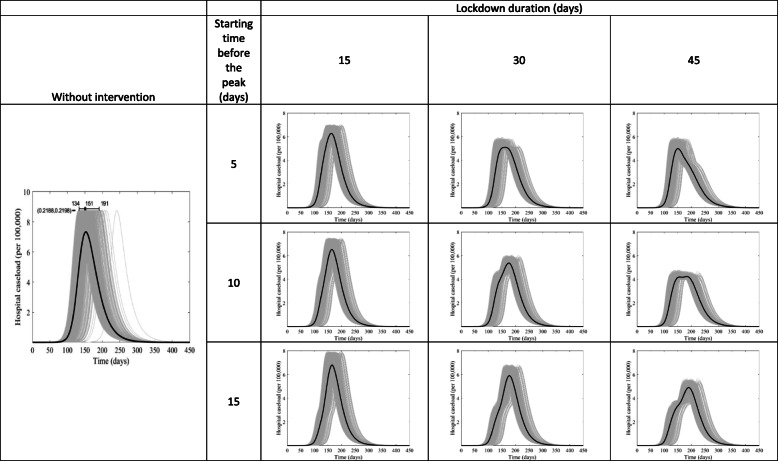
The course of the hospital COVID-19 caseload in Kuwait if the lockdown starts 5, 10, or 15 days before the peak and lasts for 15, 30, or 45 days. The uncertainty is shown in the gray shaded areas, while the solid black curve shows the mean of the simulation results

We further explored other scenarios investigating the effects of varying the timing and duration of the full lockdown on the actual infection incidence (Fig. 3) and on hospital caseload (Fig. 4). Figure 3 shows that implementation of the full lockdown 10 days before the epidemic peak and lasting for 45 days was associated with one of the best outcomes as it reduced the incidence by about 47%, and it divided the peak into two smaller ones. The same scenario of timing and duration was also highly effective in terms of reducing a hospital’s caseloads by about 46% (Fig. 4).

## Discussion

### Hypothetical lockdowns to achieve public health benefits

The model results indicated the optimal benefit in terms of reducing both attack rate and hospitalizations was achieved by implementing a full lockdown 15 days before the peak of the epidemic and for a duration of 90 days, and to a slightly lesser extent as short as 45 days. A long lockdown can be associated with severe economic and psychosocial consequences [17]. Some of these negative effects from prolonged isolation can even manifest in children and adolescents [18] as well as vulnerable populations such as immigrant workers [19]. Reported negative effects could be severe, including mental health issues, anxiety, and stigma [20], and domestic violence [21]. Indeed, the country-wide lockdown in Italy that lasted for more than two months resulted several effects that ranged from changing in children’s eating habits [22] to sleep disturbances and mental health issues such as depression and anxiety symptoms [23]. Countries look to optimize lockdown policies to achieve pandemic transmission control while at the same time avoiding major economic and socio-economic downturns. In fact, some countries such as Sweden relied on voluntary public adherence to health measures as a way to curb the pandemic [24].

The modeled results also show that implementing the full lockdown 10 days before the epidemic peak with a shorter relative duration of 45 days can still achieve a 47% reduction in hospitalizations. This may be considered a more reasonable approach as the duration still reduces the need for hospital beds, and at the same time would be associated with lower risk of negative socioeconomic and psychosocial effects. Effectiveness of the lockdown depends on the proper timing of its implementation, which should occur around 2–3 weeks before the peak of the epidemic. However, prediction of the epidemic peak is difficult, especially with new and emerging infectious diseases. Contact tracing implemented earlier in the epidemic in several countries provide better insights into the epidemic peak, which would help in gauging the perfect timing for implementation of a lockdown. For example, contact tracing can help in predicting the evolution of the COVID-19 infections so that predictions of the peak of the epidemic becomes easier [25].

Kuwait implemented a five-phase plan to gradually ease the full lockdown which was implemented from May 10 until June 30, 2020. The plan involved gradual re-opening of restaurants, cafes, shopping centers and the return to work of government employees with increasing capacity [26]. It seems that the plan didn’t result in a substantial reduction of the reported COVID-19 cases, as evidenced by the relatively stable trend of the epidemic until mid-September 2020 [27]. Kuwait is a unique country in terms of its demographics. More than 60% of Kuwait population are expatriates and the majority of those are low-skilled migrant workers [28] who live in crowded conditions [29]. These conditions increase the risk of COVID-19 transmission because large number of workers typically live in the same room and share the bathroom, kitchen and utensils. Nadoum et al. (2014) reported that 14% of migrants in his Kuwait survey had more than 14 roommates in a two-bedroom, two-bathroom apartment [30]. Furthermore, language and culture barriers could compromise effective communication of control measures to foreign workers. Stigma and fear of layoff may have prevented them from reporting any COVID-19 symptoms to their managers. Altogether, this may have contributed to the stable community transmission observed after the end of the lockdown. The Kuwaiti government implemented a set of strict intervention measures that included testing and forced institutional quarantine of arriving travelers, especially after the rapid spread of the pandemic in neighboring countries [31]. Despite these efforts, a number of clustering events have been reported especially in-migrant neighborhoods with high population density.

Data shows that after lifting the full lockdown and easing restrictions by the end of June, people resumed their daily shopping activities and migrant workers returned to their manual labor jobs. Consequently, this resulted in crowding in shopping areas, especially in high density migrant neighborhoods. The increased trend of diagnosed cases a few weeks after the lockdown was lifted supports this notion [32]. The observations described above along with our results raises the question of whether implementation of a longer lockdown could have been more effective in reducing the epidemic curve in Kuwait.

### Hypothetical lockdowns for hospital management and policy

During the first COVID-19 wave the Ministry of Health reported an increasing number of critical cases admitted to hospitals (14 in March, 52 in April, 200 in May and 112 in June) (Government of Kuwait, 2020). Implementing a well-timed 45-day lockdown could, according to the model, “flatten the curve” reducing hospitalizations by about 50% and correspondingly halving the hospital critical case load. Early reports of critical care patients estimated two-thirds of these patients required ventilation within a day of ICU admittance [5]. Halving the number of critical cases at any one time would similarly ease demand for ICU beds and ventilators by half. A 45-day lockdown has the effect of splitting the pandemic peak in to two smaller peaks while extending the overall duration of the pandemic wave. This would result in half the number of hospitalizations and critical cases with lowered ventilator demand.

## Conclusion

During the beginning of a pandemic health officials must implement an efficient contact tracing system so that exposed individuals can be quickly identified and isolated. Contact tracing can break the chains of transmission of an infectious disease and is thus an essential public health tool for controlling infectious disease outbreaks. Indeed, it has proved effective, especially during early phases of the epidemic [2]. However, once contact tracing capacity is exceeded, public health officials must shift management to employ and add other strategies to deal with community transmissions. Maintaining measures post-lockdown such as strong adherence to good hand hygiene, social distancing in public places, and mask wearing in crowded areas would be effective public health measures. Also, individuals with symptoms that resemble those of COVID-19 should avoid contacting others, especially the elderly or those with co-morbidities.

Our results show that hypothetical lockdown timing is critical for its effectiveness. Beginning a lockdown, as in the case of Kuwait, 10–15 days before the epidemic peak for 90 days (three months) was associated with the maximum reduction in attack rates of about 10% and a reduced peak of hospitalizations by nearly 60%. Even though a 90-day lockdown was found to be optimal, public health officials must consider negative impacts to the economy and mental health resulting from such a long lockdown duration. Our model shows that a lockdown of 45 days can still achieve about 50% reduction in hospitalizations. Thus, hospitals with high surge capacity (having good infrastructure, sufficient staff, stockpiles of PPE, adequate ICU, dedicated ventilators, extra cleaning schedules, and isolation rooms) may allow public health officials to opt for shorter lockdowns due to their sufficient capacity to deal with increased numbers of expected hospitalizations and critical care patients.

During the first wave of this pandemic, the major negative economic consequences resulting from implementing a country-wide lockdown raised the concept of “lockdown fatigue”, and the possibility of replacing this with local lockdown. Based on the current results, a timely implementation of a full-lockdown at local/regional scale that can be “switched on and off” and moved from on region to another depending on how the peak is progressing in each region could substantially reduce the outbreak in these “hotspots”.

## Conclusion

Experience from Kuwait and a number of other countries has revealed that border closures and air travel restrictions are not sufficient to ultimately prevent disease transmission as a way to control the pandemic. Evidence confirms that stay at home orders and other physical distancing measures including lockdowns have successfully suppressed transmission in many countries [33].

Lockdowns represent and encompass the most stringent form of social distancing with the ability to significantly reduce transmissions if timed correctly and of sufficient duration. With many countries lifting their lockdowns and returning to normality, there is a need for ongoing population-wide responsibility which is critical to reduce transmission events.

## Supplementary Information

**Additional file 1: Appendix 1**

## Data Availability

sharing is not applicable to this article as no datasets were generated or analysed during the current study.

## Abbreviations

Sars-CoV-2: Severe Acute Respiratory Syndrome Coronavirus 2: SARS-CoV-2
COVID-19: Corona virus disease-2009
SARS: Sever Acute Respiratory Syndrome
MERS CoV: Middle East Respiratory Syndrome
WHO: The World Health Organization
CTMC: stochastic Continuous-Time Markov Chain

## References

[1] Huang CL, Wang YM, Li XW, Ren LL, Zhao JP, Hu Y, Zhang L, Fan GH, Xu JY, Gu XY, Cheng Z, Yu T, Xia J, Wei Y, Wu W, Xie X, Yin W, Li H, Liu M, Xiao Y, Gao H, Guo L, Xie J, Wang G, Jiang R, Gao Z, Jin Q, Wang J, Cao B (2020). Clinical features of patients infected with 2019 novel coronavirus in Wuhan. China Lancet.

[2] Steinbrook R (2020). Contact tracing, testing, and control of COVID-19-learning from Taiwan. JAMA Intern Med.

[3] Naming the coronavirus disease (COVID-19) and the virus that causes it [https://www.who.int/emergencies/diseases/novel-coronavirus-2019/technical-guidance/naming-the-coronavirus-disease-(covid-2019)-and-the-virus-that-causes-it].

[4] Statement on the second meeting of the International Health Regulations-Emergency Committee regarding the outbreak of novel coronavirus (2019-nCoV) [https://www.who.int/news-room/detail/30-01-2020-statement-on-the-second-meeting-of-the-international-health-regulations-(2005)-emergency-committee-regarding-the-outbreak-of-novel-coronavirus-(2019-ncov) ].

[5] Mahase E (2020). Covid-19: WHO declares pandemic because of "alarming levels" of spread, severity, and inaction. BMJ.

[6] Lu N, Cheng KW, Qamar N, Huang KC, Johnson JA (2020). Weathering COVID-19 storm: successful control measures of five Asian countries. Am J Infect Control.

[7] Interventions for Community Containment. [ https://www.cdc.gov/sars/guidance/d-quarantine/app1.html].

[8] Prem K, Cook AR, Jit M (2017). Projecting social contact matrices in 152 countries using contact surveys and demographic data. PLoS Comput Biol.

[9] Otter JA, Donskey C, Yezli S, Douthwaite S, Goldenberg SD, Weber DJ (2016). Transmission of SARS and MERS coronaviruses and influenza virus in healthcare settings: the possible role of dry surface contamination. J Hosp Infect.

[10] van Doremalen N, Bushmaker T, Morris DH, Holbrook MG, Gamble A, Williamson BN, Tamin A, Harcourt JL, Thornburg NJ, Gerber SI, Lloyd-Smith JO, de Wit E, Munster VJ (2020). Aerosol and surface stability of SARS-CoV-2 as compared with SARS-CoV-1. N Engl J Med.

[11] Obray T, Tyshenko MT, Maldonado JC, Elsaadany s AWQ, Longnecker JC, Al-Zoughool M. Modeling the effect of lockdown timing as a COVID-19 control measure in countries with differing social contacts. Nat Sci Rep Submitted. 2020. 10.1038/s41598-021-82873-2 PMC7870675 33558571

[12] Mossong J, Hens N, Jit M, Beutels P, Auranen K, Mikolajczyk R, Massari M, Salmaso S, Tomba GS, Wallinga J, Heijne J, Sadkowska-Todys M, Rosinska M, Edmunds WJ (2008). Social contacts and mixing patterns relevant to the spread of infectious diseases. PLoS Med.

[13] Pellis L, Cauchemez S, Ferguson NM, Fraser C (2020). Systematic selection between age and household structure for models aimed at emerging epidemic predictions. Nat Commun.

[14] Tang B, Wang X, Li Q, Bragazzi NL, Tang S, Xiao Y, et al. Estimation of the Transmission Risk of the 2019-nCoV and Its Implication for Public Health Interventions. J Clin Med. 2020;9(2). 10.3390/jcm9020462. 10.3390/jcm9020462 PMC7074281 32046137

[15] Cao Y, Gillespie DT, Petzold LR (2006). Efficient step size selection for the tau-leaping simulation method. J Chem Phys.

[16] Allen LJ, Lahodny GE (2012). Extinction thresholds in deterministic and stochastic epidemic models. J Biol Dyn.

[17] Bonaccorsi G, Pierri F, Cinelli M, Flori A, Galeazzi A, Porcelli F, Schmidt AL, Valensise CM, Scala A, Quattrociocchi W, Pammolli F (2020). Economic and social consequences of human mobility restrictions under COVID-19. Proc Natl Acad Sci U S A.

[18] Singh S, Roy D, Sinha K, Parveen S, Sharma G, Joshi G (2020). Impact of COVID-19 and lockdown on mental health of children and adolescents: a narrative review with recommendations. Psychiatry Res.

[19] Page KR, Venkataramani M, Beyrer C, Polk S (2020). Undocumented U.S. Immigrants and Covid-19. N Engl J Med.

[20] Mackolil J, Mackolil J (2020). Addressing psychosocial problems associated with the COVID-19 lockdown. Asian J Psychiatr.

[21] Bradbury-Jones C, Isham L (2020). The pandemic paradox: the consequences of COVID-19 on domestic violence. J Clin Nurs.

[22] Di Renzo L, Gualtieri P, Pivari F, Soldati L, Attinà A, Cinelli G, Leggeri C, Caparello G, Barrea L, Scerbo F (2020). Eating habits and lifestyle changes during COVID-19 lockdown: an Italian survey. J Transl Med.

[23] Gualano MR, Lo Moro G, Voglino G, Bert F, Siliquini R: Effects of Covid-19 Lockdown on Mental Health and Sleep Disturbances in Italy. Int J Environ Res Public Health 2020, 17, 13, 10.3390/ijerph17134779. 10.3390/ijerph17134779 PMC7369943 32630821

[24] Kamerlin SCL, Kasson PM (2020). Managing COVID-19 spread with voluntary public-health measures: Sweden as a case study for pandemic control. Clin Infect Dis.

[25] García-Cremades S, Morales-García J, Hernández-Sanjaime R (2021). Improving prediction of COVID-19 evolution by fusing epidemiological and mobility data. Sci Rep.

[26] Covid-19: Kuwait lifts lockdown in all areas except Farwaniya. Gulf Business [https://gulfbusiness.com/covid-19-kuwait-lifts-lockdown-areas-except-farwaniya/ ].

[27] Kuwait [https://www.worldometers.info/coronavirus/country/kuwait/ ].

[28] PACI (2019). The public authority for civil information.

[29] Alahmad B, Kurdi H, Colonna K, Gasana J, Agnew J, Fox MA: COVID-19 stressors on migrant workers in Kuwait: cumulative risk considerations. BMJ Glob Health 2020, **5**(7), 5, 7, 10.1136/bmjgh-2020-002995. 10.1136/bmjgh-2020-002995 PMC7348320 32641292

[30] Nadoum S (2014). Geospatial Analysis for Foreign Labor force Distribution and Housing Services in Kuwait, from 2003 to 2012.

[31] Alkhamis MA, Al Youha S, Khajah MM, Ben Haider N, Alhardan S, Nabeel A, Al Mazeedi S, Al-Sabah SK (2020). Spatiotemporal dynamics of the COVID-19 pandemic in the State of Kuwait. Int J Infect Dis.

[32] COVID-19 Community Mobility Reports [https://www.gstatic.com/covid19/mobility/2020-09-30_KW_Mobility_Report_en.pdf].

[33] Islam N, Sharp SJ, Chowell G, Shabnam S, Kawachi I, Lacey B, Massaro JM, D'Agostino RB, White M (2020). Physical distancing interventions and incidence of coronavirus disease 2019: natural experiment in 149 countries. BMJ.

