# Supplementary material for: Using a stochastic continuous-time Markov chain model to examine alternative timing and duration of the COVID-19 lockdown in Kuwait: what can be done now?
Source: Arch Public Health. 2022 Jan 8;80:22. doi: 10.1186/s13690-021-00778-y (PMC8742165; doi:10.1186/s13690-021-00778-y)
Supplement: Supplementary file 1 — Additional file 1: Appendix 1 [file 13690_2021_778_MOESM1_ESM.docx]

**Appendix 1**

Using a Stochastic Continuous-Time Markov Chain Model to Examine Alternative Timing and Duration of the COVID-19 Lockdown in Kuwait: What Can be Done Now?

Mustafa Al-Zoughool *1, Tamer Oraby 2, Harri Vainio 1, Janvier Gasana 1, Joseph Longnecker 1, Walid Al Ali 3, Mohammad AlSeaidan 4, Susie Elsaadany 5 and Michael G. Tyshenko 6

Affiliations

1 Faculty of Public Health, University of Kuwait, Kuwait.

2 School of Mathematical and Statistical Sciences, University of Texas Rio Grande Valley, Edinburg, TX 78539, USA.

3 Department of Epidemiology and Biostatistics, Faculty of Public Health, University of Kuwait, Safat 13110, Kuwait.

4 Department of Occupational Health, Ministry of Health, Kuwait.

5 Department of Pathology and Laboratory Medicine, Faculty of Medicine, University of Ottawa, Ottawa, ON K1H 8M5, Canada.

6 McLaughlin Centre for Population Health Risk Assessment, Faculty of Medicine, University of Ottawa, Ottawa, ON K1N 6N5, Canada.

* Corresponding Author:

Mustafa Al-Zoughool

Faculty of Public Health, University of Kuwait, Kuwait.

Email: [mustafa.alzoughool@ku.edu.kw](mailto:mustafa.alzoughool@ku.edu.kw)

Table 1. Description of modeled transitions and their rates of the CTMC model between the SEAMHQRD-V compartments.

| **Transitions** | **Numbers in compartments that undergo changes while the rest compartments remain fixed** | **Rates (**$\boldsymbol{r}_{\boldsymbol{xy}}$**)** |
| --- | --- | --- |
| Becoming exposed (latent) | For $i=c, a,$ or $s; S_{i}\to S_{i}-1$  and $E_{i}\to E_{i}+1$ | $\beta_{i}S_{i}(\sum_{j=c,a,s} \tilde{C}_{ij}^{v}V_{j}+\sum_{j=c,a,s} \tilde{C}_{ij}\frac{A_{j}+M_{j}}{N_{j}} )$ |
| Becoming asymptomatic | For $i=c, a,$ or $s; E_{i} \to E_{i}-1$  and $A_{i}\to A_{i}+1$ | $\alpha\left( 1-p \right)E_{i}$ |
| Becoming symptomatic and mild | For $i=c, a,$ or $s; E_{i} \to E_{i}-1$  and $M_{i}\to M_{i}+1$ | $\alpha p E_{i}$ |
| Becoming symptomatic and severe | For $i=c, a,$ or $s; M_{i} \to M_{i}-1$  and $H_{i}\to H_{i}+1$ | $\gamma_{M}M_{i}$ |
| Getting quarantined (isolated) | For $i=c, a,$ or $s; M_{i} \to M_{i}-1$  and $Q_{i}\to Q_{i}+1$ | $qM_{i}$ |
| Becoming severe and hospitalized | For $i=c, a,$ or $s; Q_{i}\to Q_{i}-1$  and $H_{i} \to H_{i}+1$ | $\gamma_{Q}Q_{i}$ |
| Recovery of asymptomatic | For $i=c, a,$ or $s; A_{i} \to A_{i}-1$  and $R_{i}\to R_{i}+1$ | $\mu_{A}A_{i}$ |
| Recovery of mildly infected | For $i=c, a,$ or $s; M_{i}\to M_{i}-1$  and $R_{i}\to R_{i}+1$ | $\mu_{M}M_{i}$ |
| Recovery of severely infected | For $i=c, a,$ or $s;H_{i}\to H_{i}-1$  and $R_{i}\to R_{i}+1$ | $\mu_{H}H_{i}$ |
| Recovery of quarantined children, adults and seniors | For $i=c, a,$ or $s;Q_{i}\to Q_{i}-1$  and $R_{i}\to R_{i}+1$ | $\mu_{Q}Q_{i}$ |
| Disease-specific death of children, adults and seniors | For $i=c, a,$ or $s;H_{i}\to H_{i}-1$  and $D_{i} \to D_{i}+1$ | $\sigma_{i}H_{i}$ |
| Environmental contamination by mild and asymptomatic infected children, adults and seniors | For $i=c, a,$ or $s;$  $V_{i}\to V_{i}+1$ | $\tilde{\omega}_{A,i}A_{i}+\tilde{\omega}_{M,i}M_{i}$ |
| Environmental contamination by infected children, adults and seniors | For $i=c, a,$ or $s;$  $V_{i}\to V_{i}-1$ | $\rho V_{i}$ |

Table 2. Assumptions used in the CTMC model.

Given the uncertainties surrounding the transmission of COVID-19, a number of assumptions were made. With future validation or clarifications of these assumptions, the model can be recalibrated, and the results and findings can be revised.

| 1. Exposed individuals cannot cause infections. |
| --- |
| 1. Asymptomatic individuals are less infectious than the infected individuals, but they are making more contacts than symptomatic individuals and that makes them equal in the force of infection they are making on the susceptible population. |
| 1. Mildly infected people can only go into self-quarantine. And, severe infections can only go to hospital quarantine. |
| 1. Self-quarantined individuals can still cause some infections within the household but to a lesser degree than normal. |
| 1. We assume 100% specificity of the diagnostic tests. |
| 1. Death happens to people with specific conditions and having those conditions has a probability incorporated in the disease-specific death rate. |
| 1. Population density or travel in the model was not considered. |
| 1. The decision of people to practice social distancing and/or go into quarantine is based on the size of the epidemic which in its turn depends on the capacity of testing and reporting of case numbers. |
| 1. People cannot leave the hospital quarantine before recovery. |
| 1. Asymptomatic individuals have the recovery rate of mild individuals. |
| 1. Severely infected individuals are hospitalized without delay and so they do not contribute to either face-to-face or environmental transmission. |
| 1. The environmental contact rate is assumed to be 1/8 of the household contact rate. |

Table 3. A list of Kuwait-specific and general model parameters or variables of the CTMC model SEAMHQRD-V compartments. A description of the parameter type, its annotation, its estimated base value or range, and the evidence source reference used to establish the value are also given.

| **Type** | **Parameters**  **or Variables** | **Description** | **Base value** | **Range** | **Source/Reference** |
| --- | --- | --- | --- | --- | --- |
| Demographics | $N_{c}$ | Children’s population size  (0-18 years) | 1163769  (26.3%,) |  | The public authority for civil information. Government of Kuwait, 2019. |
|  | $N_{a}$ | Adults’ population size  (19-65 years) | 3105749  (70.3%) |  |  |
|  | $N_{s}$ | Seniors’ population size  (66+ years) | 150592  (3.4%) |  |  |
| Initial states of the epidemic | $\left( e_{0,c},e_{0,a},e_{0,s} \right)$ | Initial number of exposed individuals (children, adults, seniors) | (0,1-3,0) | 0 - 10 | Assumption:  All initial cases would be travel related from Wuhan or another infected area.  Numbers of initial cases would be small.  Assume children and seniors are not traveling to/from China or other infected areas. |
|  | $\left( a_{0,c},a_{0,a},a_{0,s} \right)$ | Initial number of infected but asymptomatic individuals (children, adults, seniors) | (0,1-3,0) | 0 - 10 | Assumption:  Travel cases appear asymptomatic (the model does not consider short term tourists traveling in to country). |
|  | $\left( m_{0,c},m_{0,a},m_{0,s} \right)$ | Initial number of mildly infected and symptomatic individuals (children, adults, seniors) | (0,1,0) | 0 or 1 | Assumption:  Mild cases undergoing travel can appear asymptomatic or near asymptomatic (the model does not consider short term tourists traveling in to country). |
|  | $\left( h_{0,c},h_{0,a},h_{0,s} \right)$ | Initial number of severely infected and symptomatic individuals (children, adults, seniors) | (0,1,0) | 0 or 1 | Assumption:  Likely any individual manifesting severe symptoms upon return would go to hospital. They would be identified initially with influenza like illness from a known area of transmission and be hospital isolated and quarantined quickly. |
| Disease-specific parameters | $\beta_{c}$ | Children infection probability upon contact with an infectious individual | 1.3 % |  | Xu Y, Li X, Zhu B, Liang H, Fang C, Gong Y, Guo Q, Sun X, Zhao D, Shen J, Zhang H, Liu H, Xia H, Tang J, Zhang K, Gong S. Characteristics of pediatric SARS-CoV-2 infection and potential evidence for persistent fecal viral shedding. Nat Med. 2020 Apr;26(4):502-505. doi: 10.1038/s41591-020-0817-4. |
|  | $\beta_{a}$ | Adults infection probability upon contact with an infectious individual | 3.5% |  | Xu Y, Li X, Zhu B, Liang H, Fang C, Gong Y, Guo Q, Sun X, Zhao D, Shen J, Zhang H, Liu H, Xia H, Tang J, Zhang K, Gong S. Characteristics of pediatric SARS-CoV-2 infection and potential evidence for persistent fecal viral shedding. Nat Med. 2020 Apr;26(4):502-505. doi: 10.1038/s41591-020-0817-4. |
|  | $\beta_{s}$ | Senior infection probability upon contact with an infectious individual | At least 3.5 % (expert opinion is that this value would be similar to that of adults) |  | Assumption:  Seniors have weaker immune systems compared to children and adults. |
|  | $\alpha$ | Rate of removal from exposed compartment (per day) (reciprocal of incubation period) | 1/5 days = 0.2 per day | Range: 3 to 7 days incubation time = 0.33-0.142 per day  [the longest time from infection to symptoms reported was 12.5 days (95% CI, 9.2 to 18)] | Guo YR, Cao QD, Hong ZS, Tan YY, Chen SD, Jin HJ, Tan KS, Wang DY, Yan Y. The origin, transmission and clinical therapies on coronavirus disease 2019 (COVID-19) outbreak - an update on the status. Mil Med Res. 2020 Mar 13;7(1):11. doi: 10.1186/s40779-020-00240-0. |
|  | $p$ | Probability of showing symptoms among those exiting the exposed compartment | 0.821 | Range: 0.5-0.9 | Mizumoto K, Kagaya K, Zarebski A, Chowell G. Estimating the asymptomatic proportion of coronavirus disease 2019 (COVID-19) cases on board the Diamond Princess cruise ship, Yokohama, Japan, 2020  Euro Surveill. 2020 Mar;25(10). doi: 10.2807/1560-7917.ES.2020.25.10.2000180.  (estimated 17.9% were asymptomatic) |
|  | $\gamma_{M}$ | Rate of progression from mild to severe infection | 1/8= 0.125  8 days*  9 days** | Range: 5–13 days  1/5-1/13  = 0.2-0.076 per day | *Huang C, Wang Y, Li X, Ren L, Zhao J, Hu Y, Zhang L, Fan G, Xu J, Gu X, Cheng Z. Clinical features of patients infected with 2019 novel coronavirus in Wuhan, China. The Lancet. 2020 Jan 24.  **Guan WJ, Ni ZY, Hu Y, Liang WH, Ou CQ, He JX, Liu L, Shan H, Lei CL, Hui DSC, Du B, Li LJ, Zeng G, Yuen KY, Chen RC, Tang CL, Wang T, Chen PY, Xiang J, Li SY, Wang JL, Liang ZJ, Peng YX, Wei L, Liu Y, Hu YH, Peng P, Wang JM, Liu JY, Chen Z, Li G, Zheng ZJ, Qiu SQ, Luo J, Ye CJ, Zhu SY, Zhong NS; China Medical Treatment Expert Group for Covid-19. Clinical Characteristics of Coronavirus Disease 2019 in China. N Engl J Med. 2020 Feb 28. doi: 10.1056/NEJMoa2002032.  Dyspnea (severe symptoms) 8 days after illness onset.  “…the mean time from illness onset to hospital admission with pneumonia was 9 days” |
|  | $\gamma_{Q}$ | Rate of progression from quarantine to severe (hospitalized) infection |  |  |  |
|  | $\mu_{M}$ ($\mu_{A}$) | Recovery rate for mildly infected (and asymptomatic) | 0.05  (20 days median)  14-37 days*  20 days** | 14-37 days  *Zhou et al. 2020 reported that viral shedding continued for a median of 20 days (maximum 37 days) in survivors and until death in non-survivors.  **Chen et al. 2020. “33% patients of the 99 infected have been discharged within 5-20 days” | Assumption:  If it is assumed that viral shedding reflects the existence of disease.  * Zhou F, Yu T, Du R, Fan G, Liu Y, Liu Z, Xiang J, Wang Y, Song B, Gu X, Guan L, Wei Y, Li H, Wu X, Xu J, Tu S, Zhang Y, Chen H, Cao B. Clinical course and risk factors for mortality of adult inpatients with COVID-19 in Wuhan, China: a retrospective cohort study. Lancet. 2020 Mar 28;395(10229):1054-1062. doi: 10.1016/S0140-6736(20)30566-3. Epub 2020 Mar 11. Erratum in: Lancet. 2020 Mar 28;395(10229):1038. Erratum in: Lancet. 2020 Mar 28;395(10229):1038.  ** Chen N, Zhou M, Dong X, Qu J, Gong F, Han Y, Qiu Y, Wang J, Liu Y, Wei Y, Xia J, Yu T, Zhang X, Zhang L. Epidemiological and clinical characteristics of 99 cases of 2019 novel coronavirus pneumonia in Wuhan, China: a descriptive study. Lancet. 2020 Feb 15;395(10223):507-513. doi: 10.1016/S0140-6736(20)30211-7. |
|  | $\mu_{H}$ | Recovery rate for severely infected | 30 days  Depends on severity.  Up to months  Minimum 20 days (range 8-37 days) (severe cases shedding virus) | Expert opinion best guess up to several weeks based on similarity to SARS coronavirus  “Duration of viral shedding ranged between 8 and 37 days. The median duration of viral shedding was 20·0 days (IQR 17·0–24·0) in survivors, but continued until death in fatal cases”* | Assumption:  Identified severe infections are under quarantine are 100% contained.  Value from Wuhan China.  “The recovery rate is over 70 per cent of the reported cases of infection in China alone”  <https://gulfnews.com/uae/coronavirus-what-does-a-covid-19-recovery-mean-for-you-1.1583824222405>.  *Zhou F, Yu T, Du R, Fan G, Liu Y, Liu Z, Xiang J, Wang Y, Song B, Gu X, Guan L, Wei Y, Li H, Wu X, Xu J, Tu S, Zhang Y, Chen H, Cao B. Clinical course and risk factors for mortality of adult inpatients with COVID-19 in Wuhan, China: a retrospective cohort study. Lancet. 2020 Mar 11. pii: S0140-6736(20)30566-3. doi: 10.1016/S0140-6736(20)30566-3. |
|  | $\sigma_{i}$; for $i=c, a,$ $s$ | Disease-specific death rate for children adults and seniors | For children: 0/31 days  For adults: <1% x 4,226= < 42.26 over 31 days Among those aged 20-54  and 1-3% for those aged 55-64  For seniors:  3-11% x 4,226 =  126.78 - 464.86  Over 31 days  Among persons aged 65-84  and 10-27%x 4226  For persons aged >=85  426- 1,141.02 over 31 days | Italy specific death rate is 7.2% (1625 deaths/22 512 cases, from Feb 20 to March 17. Not parsed by age.* | CDC updates (March 18, 2020): Severe outcomes among patients with coronavirus disease 2019-United States, February 12-March 16, 2020.  * Livingston E, Bucher K. Coronavirus Disease 2019 (COVID-19) in Italy. JAMA. 2020 Apr 14;323(14):1335. doi: 10.1001/jama.2020.4344. |
| Social mixing and contact | $C$ and $C^{H}$ | Social and household contact matrices (rates per day) | See Equation (1)  Number of contacts per day value  Singapore SARS 6.2% [95% confidence interval 3.9% to 8.6%]  China SARS  4.6%  Canada SARS  10.%  Hong Kong SARS  8% | See the entries of the matrix | The secondary household attack rate was thus low (6.2% [95% confidence interval 3.9% to 8.6%]). These findings are in contrast to the high attack rate seen in the healthcare setting (6). One possible explanation for this difference is the phase of the illness. SARS case-patients in the household tend to be in the early phase of illness whereas SARS case-patients in the healthcare settings tend to be in the later phase.    Denise Li-Meng Goh, Bee Wah Lee, Kee Seng Chia, Bee Hoon Heng, Mark Chen, Stefan Ma, and Chorh Chuan Tan. 2004. Secondary Household Transmission of SARS, Singapore. Emerg Infect Dis. 2004 Feb; 10(2): 232–234. doi: 10.3201/eid1002.030676 PMCID: PMC3322927 PMID: 15030688.    Centers for Disease Control and Prevention (CDC). Efficiency of quarantine during an epidemic of severe acute respiratory syndrome--Beijing, China, 2003. MMWR Morb Mortal Wkly Rep. 2003 Oct 31;52(43):1037-40. PMID: 14586295.    Lau JT, Lau M, Kim JH, Tsui HY, Tsang T, Wong TW. Probable secondary infections in households of SARS patients in Hong Kong. Emerg Infect Dis. 2004 Feb;10(2):235-43.    Although the household-member secondary-attack rate we found, 10.2%. One of the most important factors for household transmission was duration of exposure in the home. We found a linear association between the number of days the ill index case remained at home and the secondary attack rate.    These findings are in contrast to the high attack rate seen in the healthcare setting (6). One possible explanation for this difference is the phase of the illness. SARS case-patients in the household tend to be in the early phase of illness whereas SARS case-patients in the healthcare settings tend to be in the later phase.    Samantha D. Wilson-Clark, Shelley L. Deeks, Effie Gournis, Karen Hay, Susan Bondy, Erin Kennedy, Ian Johnson, Elizabeth Rea, Theodore Kuschak, Diane Green, Zahid Abbas, Brenda Guarda. Household transmission of SARS, 2003. CMAJ. 2006 Nov 7; 175(10): 1219–1223. doi: 10.1503/cmaj.050876. |
| Pharmaceutical, human and policy components | $r$ | Sensitivity of the diagnostic tests | 98%  The sensitivity of CT scans for COVID-19 infection)  71% (p<.001) (the sensitivity of RT-PCR for Covid19).  Italy COVID-19 cases were identified by reverse transcriptase–polymerase chain reaction (RT-PCR) testing for the severe acute respiratory syndrome coronavirus 2 (SARS-CoV-2). |  | Fang Y, Zhang H, Xie J, Lin M, Ying L, Pang P, Ji W. Sensitivity of Chest CT for COVID-19: Comparison to RT-PCR. Radiology. 2020 Feb 19:200432. doi: 10.1148/radiol.2020200432.  Onder G, Rezza G, Brusaferro S. Case-Fatality Rate and Characteristics of Patients Dying in Relation to COVID-19 in Italy. JAMA. 2020 Mar 23. doi: 10.1001/jama.2020.4683. |
|  | $n$ | Number of tested cases per day | Example country values (Italy): 8,680.14 tests per day |  | Assumption:  The number of tested cases per day is highly variable and will vary by country and time. The ability to complete testing is dependent on country diagnostic capacity, existing test kit stockpiles and healthcare infrastructure. It is assumed that a country will increase daily testing once community transmission and clusters of infections are realized.  Note that Italy, as an example country, increased its daily testing throughout March once unexpected numbes of severe infections began appearing in hospitals. Week of March 9 2020 Italy reported 60,761 tests completed (divide value by 7 days).  See: <https://www.worldometers.info/coronavirus/covid-19-testing/>. |
|  | $p_{i}\left( t \right)$  for $i= c, a,s$ | Proportion of individuals practicing social distancing as a function in time | See Equation (3)  $b_{0,i},b_{1,i},b_{2,i},$  $\tilde{p}i,$  $t_{0,i}$  $\tilde{p}_{c}=\tilde{p}_{s}=1$, $\tilde{p}_{a}=.5$ |  |  |
|  | $q$ | Probability of mildly infected going into self-quarantine | 0.9 | () | Assumption:  High compliance, very few individuals will disregard public health officials.  SARS expert estimate: First travel related patients and contact tracing by public health officials would place all identified people in “home quarantine”. Public health would seek to identify 100% of contacts and first cases would be self-quarantined. |
|  | $C^{V}$ | Contact rates with environment | $\frac{1}{8} C^{H}$ |  | Assumption:  Expert opinion estimates the contact rate with the environment to be 1/8^th^ the household contact rate or 3 hours each day as individuals leave the household to complete shopping and errands. |
| Environmental  variables | $\omega_{A}$ | Number of individuals equivalent to environmental contamination/deposit made by asymptomatic individual per place | 1 |  |  |
|  | $\omega_{M}$ | Number of individuals equivalent to environmental contamination/deposit made by mildly infected individual per place | 1 |  |  |
|  | $\rho$ | Natural removal rate of the environmental contamination | 1/3 |  |  |
|  | $K$ | Cleaning rate of the environmental contamination | 0 |  |  |
|  |  |  |  |  |  |
